# Noninvasive Measurement of Murine Hepatic Acetyl-CoA ^13^C-Enrichment Following Overnight Feeding with ^13^C-Enriched Fructose and Glucose

**DOI:** 10.1155/2013/638085

**Published:** 2013-06-10

**Authors:** Filipa Carvalho, Joao Duarte, Ana Rita Simoes, Pedro F. Cruz, John G. Jones

**Affiliations:** ^1^Center for Neurosciences and Cell Biology, Department of Zoology, University of Coimbra, Coimbra 3004-517, Portugal; ^2^Advanced Imaging Research Center, University of Texas Southwestern Medical Center, Dallas, TX 75390-8568, USA; ^3^Department of Chemistry, University of Coimbra, Coimbra 3004-535, Portugal; ^4^APDP-Portuguese Diabetes Association, Lisbon 1250-261, Portugal

## Abstract

The ^13^C-isotopomer enrichment of hepatic cytosolic acetyl-CoA of overnight-fed mice whose drinking water was supplemented with [U-^13^C]fructose, and [1-^13^C]glucose and *p*-amino benzoic acid (PABA) was quantified by ^13^C NMR analysis of urinary *N*-acetyl-PABA. Four mice were given normal chow plus drinking water supplemented with 5% [1-^13^C]glucose, 2.5% [U-^13^C]fructose, and 2.5% fructose (Solution 1) overnight. Four were given chow and water containing 17.5% [1-^13^C]glucose, 8.75% [U-^13^C]fructose and 8.75% fructose (Solution 2). PABA (0.25%) was present in both studies. Urinary *N*-acetyl-PABA was analyzed by ^13^C NMR. In addition to [2-^13^C]- and [1,2-^13^C]acetyl isotopomers from catabolism of [U-^13^C]fructose and [1-^13^C]glucose to acetyl-CoA, [1-^13^C]acetyl was also found indicating pyruvate recycling activity. This precluded precise estimates of [1-^13^C]glucose contribution to acetyl-CoA while that of [U-^13^C]fructose was unaffected. The fructose contribution to acetyl-CoA from Solutions 1 and 2 was 4.0 ± 0.4% and 10.6 ± 0.6%, respectively, indicating that it contributed to a minor fraction of lipogenic acetyl-CoA under these conditions.

## 1. Introduction

Hepatic acetyl-CoA is a central metabolite that links the major catabolic and anabolic pathways of hepatic carbohydrate and lipid metabolism. These pathways are normally highly regulated over the fasted and fed states and are central to glucose and lipid homeostasis. In the setting of overnutrition where caloric intake exceeds whole body energy demands, there is a remodelling of hepatic carbohydrate and lipid metabolism that is characterized in part by changes in catabolic and anabolic acetyl-CoA fluxes. In both animals and humans, the excessive consumption of sucrose or high fructose corn syrup may modify hepatic acetyl CoA fluxes since fructose is rapidly metabolized by the liver to triose phosphates that in turn are potential sources of acetyl-CoA. The increased acetyl-CoA availability coupled with an upregulation of lipogenic enzymes is implicated in the characteristic accumulation of hepatic triglycerides following sucrose or fructose feeding [1–4]. Therefore, there is renewed interest in quantifying the contributions of dietary fructose and other precursors to the hepatic acetyl-CoA pool using stable isotope tracer methods. Given that hepatic acetyl-CoA may be derived from a wide range of substrates, these assays need to be as informative as possible. NMR offers an advantage in that it can resolve multiply-enriched ^13^C-species, or isotopomers of metabolites derived from a mixture of ^13^C-enriched substrates. In the case of acetyl-CoA, enrichment from up to four different ^13^C-labeled substrates may be resolved on the basis of the 2^2^ acetyl isotopomer permutations. However, *in vivo* NMR observation of hepatic acetyl-CoA enrichment is not possible because of its low tissue abundance. Moreover, its analysis from liver extracts by NMR is compounded by its high molecular weight and poor stability. As a result, acetyl-CoA enrichment is inferred from the analysis of downstream metabolites such as glutamate and glutamine, whose carbons 4 and 5 are descended from those of acetyl-CoA. However, for the *in situ* liver, a significant fraction of hepatic glutamate and glutamine may be derived from extrahepatic tissues [5]; hence, the carbon 4 and 5 enrichments may not accurately reflect the true hepatic acetyl-CoA isotopomer distribution.

Hellerstein et al. pioneered a noninvasive method for sampling the enrichment of hepatic acetyl-CoA from stable-isotope tracers by utilizing the hepatic acetylation pathways for xenobiotic agents such as sulfamethoxazole [6]. These compounds are acetylated by acetyl-CoA followed by rapid clearance into the urine. Hepatic acetyl-CoA enrichment is then inferred by analysis of the acetylated compounds in the urine. These methods were developed for gas-chromatography-mass spectrometry (GC-MS) analyses and yielded information on acetyl-CoA production from ^13^C-precursor substrates. To our knowledge, this elegant approach has yet to be translated into ^13^C-NMR isotopomer analysis. 

*p*-Amino benzoic acid (PABA) is an attractive candidate for acetyl-CoA chemical biopsy. It is a widely used, though nonessential nutritional supplement in humans. Following ingestion, it is cleared rapidly from the body via the urine and is thus routinely used as a marker for assessing the completeness of 24-hour urine collection [7]. The majority of PABA is rapidly acetylated in the liver to form *N*-acetyl PABA, followed by rapid clearance into urine [8]. The acetylation of PABA by *N*-acetyl transferase utilizes a pool of cytosolic hepatic acetyl-CoA [9]. Thus, enrichment of *N*-acetyl PABA from ^13^C-enriched precursor substrates potentially provides a means of determining their contribution to *de novo* lipogenesis, which also utilizes cytosolic acetyl-CoA. There is high current interest in understanding the role of specific substrates such as fructose in sustaining *de novo* lipogenesis, but there has been little progress in quantifying their contributions to the cytosolic acetyl-CoA precursor pool. 

In contrast to oxidative tissues such as heart and muscle, where under most conditions, specific acetyl-CoA ^13^C-isotopomers can be related to the catabolism of specific ^13^C-enriched oxidative precursor substrates [10, 11], hepatic acetyl-CoA is also extensively labeled from Krebs cycle intermediates via pyruvate recycling. Pyruvate recycling is defined as the exchange of pyruvate with Krebs cycle intermediates via pyruvate carboxylase-phosphoenolpyruvate carboxykinase-pyruvate kinase and/or malic enzyme activities [12] followed by its oxidation to acetyl-CoA. This process generates isotopomers from randomized Krebs cycle intermediates where the parent ^13^C-enriched substrates cannot be identified.

In this paper, we demonstrate that ^13^C NMR analysis of urinary *N*-acetyl-PABA can be used to noninvasively measure the contribution of ^13^C-enriched fructose and other dietary precursors to the lipogenic acetyl-CoA pool in mice during a normal nocturnal feeding interval. The analysis also reveals the presence of pyruvate cycling and its effects on measuring the contributions of various ^13^C-enriched precursor substrates to hepatic cytosolic acetyl-CoA production. 

## 2. Methods

[1-^13^C]glucose at 99% enrichment was obtained from Isotec, Miamisburg, OH, USA, and [U-^13^C]glucose at 99% enrichment was obtained from Cambridge Isotopes, Andover, MA, USA.

### 2.1. Animal Studies

The animal study protocol conformed to the NIH guide and the Institutional guidelines for the Care and Use of Laboratory Animals and was approved by the Local Ethics Committee. Four Male C57/Bl6 mice weighing 25 ± 8 g were housed in a 12 h light/12 h dark cycle (lights on from 07 : 00.19 : 00) with free access to food (standard chow 60% carbohydrate, 16% protein, and 3% lipid) and water. At 09:00 on day 1, the drinking water was supplemented with 5% w/w of unlabeled fructose, and glucose and 2.5 mg/L of PABA. At 19:00 on day 3, the drinking water was replaced with one containing 5% [1-^13^C]glucose, 2.5% [U-^13^C]fructose, 2.5% unlabeled fructose and 2.5 mg/L PABA. To determine the effects of increased sugar concentrations on acetyl-CoA sources, a second set of four mice were presented with higher glucose and fructose concentrations in the drinking water corresponding to 35% w/w sucrose (i.e., 17.5% [1-^13^C]glucose, 8.75% [U-^13^C]fructose and 8.75% unlabeled fructose)—a concentration that is widely used to induce steatosis in rodents. The PABA concentration was maintained as for the first study. For each animal, the urine produced throughout the overnight period was pooled.

### 2.2. N-Acetyl-PABA Purification and Processing

Urine samples were centrifuged to remove impurities and the pH was adjusted to 7, to prevent hydrolysis reactions. Following evaporation at 37°C, samples were further purified by solid phase extraction. A 2-gram DSC-18 column (Sigma-Aldrich) was prewashed with 3.4 mL methanol followed by 6.6 mL of water acidified to pH 4.0 with trifluoroacetic acid (TFA)—hereafter referred to as acidic water. The dried urine was dissolved in 2 mL of water, the solution pH was adjusted to 2.5 with TFA, and the solution was applied to the column. The column was washed with 8 mL of acidic water, followed by 8 mL of 10% methanol/90% acidic water. *N*-Acetyl-PABA was eluted with 8 mL of 50% methanol/50% acidic H_2_O and the fraction evaporated to dryness. For ^13^C NMR spectroscopy, the residue was then dissolved in 0.6 mL of 99.9% ^2^H_2_O and the pD was adjusted to 7.0 with 2 M sodium carbonate.

### 2.3. NMR Spectroscopy

Proton-decoupled ^13^C NMR spectra of *N*-acetyl PABA were acquired at a temperature of 25°C with a Bruker Avance III 400 spectrometer (static magnetic field = 9.4 T), equipped with a 5 mm BBFO broadband probe. An acquisition time of 2.7 seconds and pulse delay of 2.0 seconds were used following a 30-degree flip angle pulse. Before Fourier transformations ^13^C-NMR spectra were multiplied by a 0.5 Hz Lorentzian function. The number of free induction decays that were collected for each ^13^C spectrum was 3536 (4.6 hours of collection time).

### 2.4. Quantification of Acetyl ^13^C Excess Enrichments

The total ^13^C-enrichment of carbon 2 from the acetyl moiety of *N*-acetyl PABA was calculated by comparing the total multiplet area the carbon signal with the mean areas of the pair of aromatic PABA signals resonating at 130.85 ppm (C2, C6) and 121.57 (C3, C5) after correction for stoichiometry, T_1_ and nOe effects. A correction factor was obtained by quantifying the ratio of carbon 2 to aromatic PABA signal intensities from a ^13^C spectrum of nonenriched *N*-acetyl PABA acquired under identical conditions.

(1) Total  carbon  2  enrichment  (%)  =Total  carbon  2  AreaPABA  signal  area×CF×1.11×100.

Total carbon 2 ^13^C-enrichment was resolved into its component [2-^13^C]- and [1,2-^13^C_2_]acetyl isotopomers by multiplying with the ratio of carbon 2 singlet (C2S) and carbon 2 doublet (C2D) components relative to the total carbon 2 resonance area (C2) as follows

(2) total  [2-C13]acetyl  enrichment(%)  =C2SC2×total  carbon  2  enrichmenttotal  [1,2-C132]acetyl  enrichment  (%)  =C2DC2×total  carbon  2  enrichment.

The [2-^13^C]acetyl isotopomers represent the sum of back ^13^C enrichment of carbon 2 and excess enrichment due to [2-^13^C]acetyl-CoA derived from metabolism of the ^13^C-enriched substrates. Excess enrichment from [2-^13^C]acetyl-CoA was calculated by subtracting the natural abundance level (1.11%) from the total [2-^13^C]acetyl enrichment. Consider

(3) excess  [2-C13]acetyl  enrichment  (%)  =  total  [2-C13]acetyl  enrichment−1.11.

Background levels of [1,2-^13^C_2_]acetyl are insignificant compared to contributions from [1,2-^13^C_2_]acetyl CoA; hence, no correction was applied to the measured [1,2-^13^C_2_]acetyl enrichment. The carbon 1 signal also consists of a singlet (C1S) and doublet (C1D). The doublet is derived from the common [1,2-^13^C_2_]acetyl isotopomer and therefore represents the same enrichment level. On this basis, the total enrichments of carbon 1 and its constituent [1-^13^C]isotopomer were calculated as follows:

(4) total  carbon  1  enrichment  (%)  =total  [1,2-C132]acetyl  enrichment×C1C1D,total  [1-C13]acetyl  enrichment  (%)  =C1SC1×total  carbon  1  enrichment,excess  [1-C13]acetyl  enrichment  (%)  =total  [1-C13]acetyl  enrichment−1.11.

### 2.5. Statistics

All results are presented as means ± standard error and comparisons were made by an unpaired *t*-test (two tailed) performed using Microsoft Excel. Statistical significance was considered as a *P* value of < 0.05.

## 3. Results

### 3.1. Recovery and ^13^C NMR Isotopomer Analysis of N-acetyl PABA

**Figure 1:**
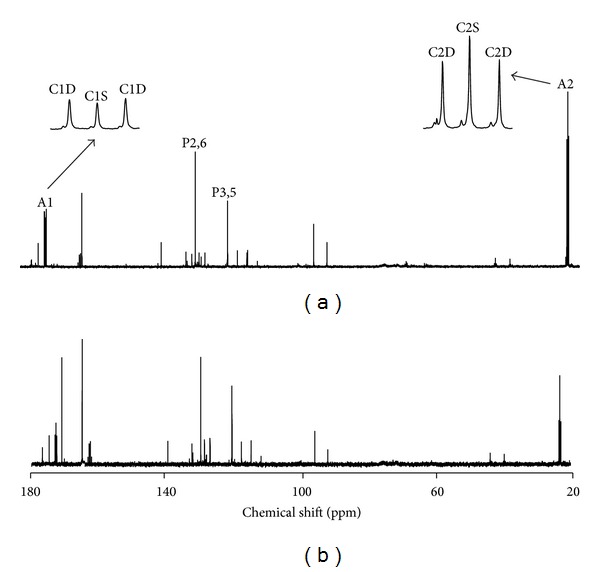
^13^C NMR spectra of urinary *N*-acetyl PABA derived from mice whose drinking water was supplemented with 8.75% [U-^13^C]fructose/8.75% unlabeled fructose/17.5% [1-^13^C]glucose (a) and 2.5% [U-^13^C]fructose/2.5% unlabeled fructose/5% [1-^13^C]glucose (b). Spectra are scaled to the *N*-acetyl PABA aromatic ^13^C-signals of the 2,6 ring carbons (P2,6) and the 3,5 ring carbons (P3,5). For spectrum A, the *N*-acetyl resonances of carbon 1 (A1) and carbon 2 (A2) are shown in expanded form featuring the singlet (C1S, C2S) and doublet (C1D, C2D) components. Signals from *N*-acetyl-*p*-amino hippuric acid can be seen as small shoulders to the left-hand side of each of the carbon 1 and 2 multiplet components.

The 0.25% PABA present in the drinking water was well tolerated by the mice. Their water intake was estimated to be 15 mL per animal per night, within the normal range for mice. Following urine harvesting and SPE, 14 ± 2 *μ*mol of *N*-acetyl PABA were recovered for ^13^C NMR analysis. *N*-Acetyl PABA was the most abundant of the PABA metabolites, but also present were *p*-amino hippurate (PAH) and minor amounts of *N*-acetyl-*p*-amino hippurate (*N*-acetyl-PAH), representing 5%–10% of *N*-acetyl PABA levels. The ^13^C NMR acetyl signals of *N*-Acetyl-PAH were proximal to those of *N*-acetyl PABA but did not preclude precise quantification of the ^13^C spin-coupled multiplets (see Figure 1). 

**Figure 2:**
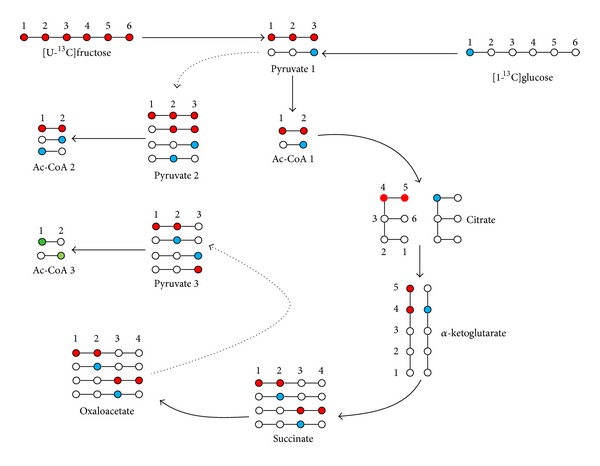
Schematic of acetyl-CoA isotopomer formation from [1-^13^C]glucose (blue filled circles) and [U-^13^C]fructose (red filled circles). For simplicity, unlabeled isotopomers are not shown. Conversion of the substrates to pyruvate generates a pair of pyruvate isotopomers (Pyruvate 1), and oxidation by pyruvate dehydrogenase yields a pair of acetyl-CoA isotopomers (Ac-CoA 1). Conversion of the substrates to pyruvate followed by complete cycling of pyruvate via Krebs cycle intermediates (oxaloacetate-malate-fumarate), represented by the dashed arrow, yields another set of pyruvate and acetyl-CoA isotopomers designated Pyruvate 2 and Ac-CoA 2. Isotopomers of Krebs cycle intermediates formed by incorporation of [1,2-^13^C_2_]acetyl CoA and [2-^13^C]acetyl-CoA into the cycle also generate pyruvate and acetyl-CoA isotopmers via pyruvate cycling (pyruvate 3 and Ac-CoA 3). Unlike the previous cases, the two acetyl-CoA isotopomers (shown in green) cannot be traced to their parent ^13^C-substrates. The arrangement of pyruvate and acetyl-CoA into three groups is intended purely for illustrating isotopomer formation and does not imply compartmentation of these pools *in situ*.

^13^C NMR spectra of the purified urine extract revealed C1 and C2 acetyl resonances consisting of singlets flanked by doublets with a common coupling constant of 51.0 Hz, characteristic of ^13^C-^13^C coupling between methyl and carboxyl carbons. Analysis of the carbon 2 resonance revealed excess enrichments from both [1,2-^13^C_2_]acetyl and [2-^13^C]acetyl-CoA, shown in Table 1, indicative of contributions of acetyl-CoA generated from metabolism of both [U-^13^C]fructose and [1-^13^C]glucose. The two natural-abundance ^13^C-signals from the PABA aromatic moiety used as intramolecular ^13^C-enrichment standards had signal areas that were within 5% of each other but the C3,5 signal line width was consistently broader than that of C2,6; hence, it appeared as a less intense peak in the ^13^C NMR spectrum (see Figure 1). From the analysis of ^13^C-aromatic and acetyl ^13^C-resonance intensities of *N*-acetyl PABA, estimates of excess acetyl ^13^C-enrichments were surprisingly low given the amount of sugar that had been consumed indicating that the bulk of acetyl-CoA (>80%) had originated from unlabeled dietary or endogenous substrates (see Table 1). In the absence of pyruvate cycling fluxes, [1,2-^13^C_2_]acetyl and [2-^13^C]acetyl-CoA are the only isotopomers that can be generated from the glycolytic metabolism of [U-^13^C]fructose and [1-^13^C]glucose (see Figure 2). However, pyruvate cycling activity results in the generation of [1-^13^C]acetyl-CoA (see Figure 2). This isotopomer was present in significant amounts relative to [2-^13^C]acetyl and [1,2-^13^C_2_]acetyl, indicating that a significant fraction of acetyl-CoA enrichment was derived via pyruvate cycling activity. 

## 4. Discussion

The sampling of hepatic metabolic intermediates by chemical biopsy is a well-established method for analyzing isotopic labeling of hepatic metabolic intermediates in both humans [6, 13–20] and rodents [21–23]. This approach has been widely used for the study of hepatic glucose and metabolism but has been less applied for acetyl-CoA and lipid synthesis. The main reasons include (1) the development of techniques based on product mass isotopomer distribution analysis (MIDA) and deuterated water that circumvent the need for measuring the hepatic acetyl-CoA precursor enrichment [6, 24, 25] and (2) reported uncertainties in the measurement of cytosolic acetyl-CoA enrichment from metabolic tracers by chemical biopsy. In perfused rat livers, cytosolic acetyl CoA sampled by different acetylation probes yielded different ^13^C-enrichment profiles of acetyl-CoA suggesting that cytosolic acetyl-CoA was not homogeneously enriched from ^13^C-precursor substrate [9]. On the other hand, for humans infused with ^13^C-acetate, the acetyl-CoA precursor enrichment predicted by MIDA of VLDL-triglyceride matched that measured by chemical biopsy using sulfamethoxazole [6]. To our knowledge, this remains the only study that has directly compared chemical biopsy and MIDA assays of lipogenic acetyl-CoA enrichment. However, it remains to be seen if this relationship is true for other ^13^C-precursor substrates, including fructose, and for different animal species. 

Our data suggest that compared to glucose, fructose contributes more to cytosolic acetyl-CoA appearance. However, its contributions are relatively minor compared to those of unlabeled endogenous sources. Human studies have shown that only a very minor fraction (<1%) of ingested fructose is converted into lipids over the ensuing 6 hours [26]. For naturally feeding mice presented with fructose in their drinking water overnight, the contribution of this substrate to *de novo* lipogenesis is not known but its limited appearance in acetyl-CoA suggests that it is also a minor contributor of carbons for lipid synthesis during overnight feeding. 

For determining the contributions of ^13^C-enriched precursor substrates to hepatic acetyl-CoA appearance, pyruvate cycling activity has important implications. In the absence of pyruvate cycling, acetyl-CoA isotopomers that are formed from the metabolism of ^13^C-enriched precursors can be unambiguously identified, for example, [1-^13^C]glucose metabolism generates [2-^13^C]acetyl-CoA plus an unlabeled acetyl-CoA while [U-^13^C]fructose metabolism yields two [1,2-^13^C_2_]acetyl-CoA molecules. On this basis, the relative contributions of these substrates to acetyl-CoA formation may be directly determined from the ratio of [2-^13^C]- to [1,2-^13^C_2_]acetyl enrichments. With pyruvate cycling, the source of [2-^13^C]acetyl-CoA is no longer exclusively derived from [1-^13^C]glucose metabolism, but may also be indirectly generated from [U-^13^C]fructose following entry of [1,2-^13^C_2_]acetyl-CoA into the Krebs cycle, as shown in Figure 2. Moreover, for [1-^13^C]glucose metabolism, pyruvate cycling results in the randomization of ^13^C into both carbons of acetyl-CoA resulting in a mixture of [1-^13^C]- and [2-^13^C]acetyl-CoA isotopomers. For [U-^13^C]fructose, or indeed any other precursor that generates [U-^13^C]- or [2,3-^13^C_2_] pyruvate, randomization via pyruvate cycling does not generate alternative isotopomers to [1,2-^13^C_2_]acetyl-CoA. Moreover, under conditions of low excess ^13^C-enrichments, [1,2-^13^C_2_]acetyl-CoA cannot be formed from the recycling of first-pass Krebs cycle isotopomers. Therefore, for [U-^13^C]fructose, the analysis of [1,2-^13^C_2_]acetyl-CoA enrichment reveals its contributions to acetyl-CoA flux regardless of pyruvate cycling activity. In contrast, for determining the precise contribution of [1-^13^C]glucose to acetyl-CoA flux, pyruvate cycling and Krebs cycle fluxes need to be known. The information content provided by the four possible ^13^C-isotopomers of acetyl-CoA is insufficient for describing each of these flux parameters. In comparison, the information content of glutamate ^13^C-enrichment where up to 32 different ^13^C-isotopomers can be generated is sufficient to describe pyruvate cycling, Krebs cycle, and acetyl-CoA fluxes [27–29]. Upper and lower limits for the contribution of [1-^13^C]glucose to acetyl-CoA production can be postulated based on the sources of [1-^13^C]acetyl-CoA excess enrichment. If [1-^13^C]acetyl-CoA had been entirely derived from pyruvate cycling of [3-^13^C]pyruvate, then at this limit, the true contribution of [1-^13^C]glucose is related to the sum of [1-^13^C]- and [2-^13^C]acetyl-CoA enrichments. On the other hand, if all the observed [1-^13^C]acetyl-CoA was indirectly derived from [U-^13^C]fructose, the true contribution of [1-^13^C]glucose to acetyl-CoA flux is related to the difference of [1-^13^C]- and [2-^13^C]acetyl-CoA enrichments. Modelling studies with tcaSIM (tcaSIM is available from http://www.utsouthwestern.edu/utsw/home/research/AIRC). indicate that about 50% of [1-^13^C]acetyl-CoA is derived from cycling of [3-^13^C]pyruvate with the remaining contribution derived from randomized Krebs cycle isotopomers. In this case, the estimated acetyl-CoA contribution from [1-^13^C]glucose is approximately halfway between the upper and lower limits, that is, 2.2% and 5.0% for the 5%-5% and 17.5%-17.5% solutions, respectively.

In summary, PABA administered in the drinking water can be used to sample hepatic acetyl-CoA enrichment in naturally feeding mice presented with ^13^C-enriched glucose and fructose. The urinary *N*-acetyl-PABA can be analyzed by ^13^C NMR and all four acetyl-CoA ^13^C-isotopomers can be resolved and quantified. Pyruvate cycling introduces substantial uncertainties in estimating the contribution of singly labeled substrates that are metabolized to [2-^13^C] or [3-^13^C]pyruvate, such as [1-^13^C]glucose, but does not influence estimates from ^13^C-enriched substrates that generate [U-^13^C]pyruvate. PABA isotopomer analysis reveals a low contribution of exogenous [U-^13^C]fructose to hepatic acetyl-CoA synthesis during overnight feeding.

## Figures and Tables

**Table 1:** Hepatic acetyl-CoA ^13^C-isotopomer abundances and fractional contributions of substrates to hepatic acetyl-CoA appearance in naturally feeding mice with their drinking water supplemented by two different concentrations of a 99% [1-^13^C]glucose/50% [U-^13^C]fructose mixture. *The estimated glucose contributions represent upper and lower limits based on the assumption that [1-^13^C]acetyl CoA was either entirely derived from [1-^13^C]glucose or from [U-^13^C]fructose, respectively.

Drinking water sugar levels (% w/v)	Acetyl ^13^C-isotopomer excess enrichments			Substrate contributions to acetyl CoA (%)		
	[1-^13^C]acetyl	[2-^13^C]acetyl	[1,2-^13^C_2_]acetyl	Glucose*	Fructose	Endogenous
5% Fructose and 5% glucose (*n* = 4)	0.4 ± 0.1	1.1 ± 0.1	2.0 ± 0.2	3.0 ± 0.2	4.0 ± 0.4	93.0 ± 0.4
				1.4 ± 0.1	4.0 ± 0.4	94.6 ± 0.4

17.5% Fructose and 17.5% glucose (*n* = 4)	1.1 ± 0.1	2.6 ± 0.1	5.3 ± 0.3	7.4 ± 0.2	10.6 ± 0.6	82.2 ± 0.4
				3.0 ± 0.1	10.6 ± 0.6	86.6 ± 0.4
